# What Do You Know about Amalgam

**Published:** 1889-03

**Authors:** 


ARTICLE IV.

### WHAT DO YOU KNOW ABOUT AMALGAM.

Dr. A. H. Fuller then read an interesting paper on "What do we Know about Amalgam," the discussion of which was opened by Dr. Patterson, who said that this subject was apt to cause much discussion. He did not agree with all the conclusions of the writer. In regard to the injurious effects. He does not believe there can be any injurious effect after the filling is hardened and oxides are formed. As he inserts an amalgam filling, there is no change, no shrinkage, no expansion. All cavities that are to be filled with amalgam should be made simple cavities, with three or four walls, using matrix if necessary. He uses a mallet, or other force, with a pad of cotton or spunk under point of instrument, and inserts it as rapidly as possible to make a solid filling. If the cavity is properly prepared, there is no thin edge to break off.

In regard to amalgams in general, he objects to the color in the mouth in any position, and only uses it on account of the pecuniary consideration. Some dentists assert that they will not use it, or anything but the very best material—that is gold; but in the country, and where it is necessary to earn a living, it is necessary to use this material, though he constantly uses the cement more and amalgam less for this class of work.

He has had no particular experience with copper amalgam, but objects to it more than the others on account of the color. Can not see how copper amalgam could have any poisonous effect, as it oxidizes, and the oxide is inert since there can not be sufficient acetic acid in the mouth to act on the same.

Dr. Stevens is obliged to use amalgam often in his practice. In building up a tooth with this material, he sometimes makes a band of gold, which is fitted perfectly to the root and then filled with amalgam.

Dr. Patterson asks that statements, in regard to cases where amalgam is supposed to be injurious, be substantiated, as we have no positive proof that the trouble ascribed by many to the mercury in fillings, is due to it.

Dr. Pierce is thankful to Dr. Fuller for his paper, as it permits him to bring out one or two points on the subject of which he has made a study. He doubts that there is any injurious effect from amalgam fillings after hardening, as he has made the test repeatedly with gold foil and found no traces of the fumes of mercury. Before the filling is hardened, there are fumes that affect gold foil, but when once crystalized, it will not affect it. As to the poisonous effects of the copper, if mercury and copper form no chemical compound, and if the copper is only held by suspension, there will be copper salts formed by the acidulated moisture in the mouth, and these salts are poisonous. Put a copper plate in acidulated water and it will be oxidized, but the copper salts continue to be deposited. But, if mercury and copper do make a chemical compound then it is possible this compound may be of a different character from either mercury or copper. He has experimented with all amalgams and finds copper salts in every amalgam containing copper. He disagrees with Dr. Fuller when he says that the dryer the amalgam the more shrinkage there will be, as he has found that the dryer the amalgam, as long as it can be handled, the better. As to the toxical effects of copper salts, he is an advocate of the idea that the finer you divide, the more effect there is, as he proves in the use of mercury, of which a teaspoonful may be taken without producing any effect, but if there is sufficient warmth of body to produce vapor from it, there will be a poisonous effect.

Dr. Conrad has never seen a single case where there were any bad constitutional effects from amalgam fillings. If there is a liberation of free copper for formation of copper salts in the mouth, there would, in time, be an appreciable loss of substance, which is not found to be the fact. The use of amalgam is dangerous, because the copper amalgam makes such a perfect filling that we are liable to put in too many; that old copper amalgams look like gold. Copper amalgam will not discolor tooth substance, makes a perfect edge, and that, in the case of children with soft teeth, makes the best filling.

Dr. Patterson said that it has not been shown yet that there are poisonous effects from copper amalgam *in the mouth*, but only that there are such effects out of the mouth, which no one denies. Nitric acid will destroy a tooth out of the mouth, but, though nitric acid exists in the mouth, we know it does not destroy the tooth in the mouth. Nitro-muriatic acid will dissolve gold, but, though it exists in the mouth, we know it does not dissolve the gold filling in the mouth; and it is not proved yet that there is any poisonous effect from the copper salts in the mouth.

Dr. Pierce said it is difficult to prove any fact or effect in the mouth, and it is impossible to get positive evidence in the mind of a doubting Thomas. If you touch an amalgam filling in the mouth with acetic acid, it causes an immediate flow of saliva from Wharton's duct; and that while this might be something else, he believes it to be the irritation caused by the poison salts.

Dr. Patterson said the action of acetic acid on the gustatory nerves alone, would cause the flow of saliva.

Dr. Conrad said that as to amalgam fillings which were as green as grass, he had seen a tooth filled with gold which was almost as green as yellow grass, but did not know what caused it

Dr. Harper said that in regard to discoloration of copper amalgam he had found that in some mouths it does not discolor at all; in others, slightly, and in others was very dark. In regard to green color, he had transplated a tooth, filled the root with gutta-percha, and the tooth turned green.

Dr. McMillen told of a tooth crowned which turned green.

Dr. Morrison recommended the use of the French bibulous paper to use in packing amalgam.

Dr. Fuller said that Tomes gives careful evidence that he has not seen evil effects from amalgam fillings—and many others also—but there are many who have given evidence to the contrary, and we should accept this in preference to the other. Because one man has not seen a thing, does not prove that it does not exist. Some constitutions are possessed of certain idiosyncrasies, and certain articles that are harmless to the majority of people, act as poison to them, as, for instance, strawberries, and again, honey, and others; and some people have this idosyncrasy that their system will not admit of the presence of even the smallest amount of mercury. He then gave two cases where there was every evidence of poisoning from the mercury in an amalgam filling; one where it was used for setting a crown, and the other as a filling in the mouth of a boy, and concluded that, "there being evidence in favor of it, the evidence to the contrary should not be accepted in preference."

Dr. Patterson said that Dr. Fuller has just given the first authentic case of injury, due to mercury in amalgam, that he has ever heard; but even this being the case, to what point does the discussion tend? Are we to do without amalgam because there are a few cases of injury from its use? Every physician will tell you that he meets with idosyncrasies in people, some who can not endure morphine or quinine, etc., but they do not, on that account, discontinue the use of these valuable remedies.

Dr. Fuller wished to correct any such impression, and moved the subject be passed.—*Archives of Dentistry.*

